# Health literacy and chronic disease: a comparison of somatic and mental illness

**DOI:** 10.3389/fpubh.2025.1523723

**Published:** 2025-02-18

**Authors:** Lennert Griese, Doris Schaeffer

**Affiliations:** ^1^School of Public Health, Bielefeld University, Bielefeld, Germany; ^2^Hertie School, Berlin, Germany

**Keywords:** health literacy, health information, chronic illness, mental health, health care, prevention, health promotion

## Abstract

**Background:**

Health literacy (HL) is increasingly recognized as essential for preventing and managing chronic illness but also for strengthening health resources and skills. However, studies on HL of people with chronic illness that adopt a multidimensional approach encompassing the three HL domains health care, disease prevention, and health promotion, remain scarce. This study aims to (a) compare HL across these three domains in individuals with chronic somatic illness, chronic mental illness and those without any chronic illness, (b) to explore where difficulties in managing health-related information occur and how these differ between groups, and (c) to analyze the relationship between demographic, social, and psychological factors and HL.

**Methods:**

Data from a quantitative cross-sectional survey in Germany were stratified according to respondents with at least one chronic somatic illness, at least one chronic mental illness and without chronic illness. The survey was conducted by means of paper-assisted personal interviews. HL was measured in three domains health care, disease prevention and health promotion. Age, educational level, social status, financial resources, number of chronic illnesses, social support, and self-efficacy were included in the analysis as potential determinants of HL. Differences between groups were analyzed using bivariate statistics; multiple linear regressions were calculated to examine relationships between potential determinants and HL.

**Results:**

Respondents with chronic mental illness showed lowest HL, followed by those with chronic somatic illness. Respondents without chronic illness achieved highest HL. This pattern was consistent across all three HL domains. Among all groups, HL was lowest in the domain of health promotion. Notable differences emerged in perceived difficulties, with respondents with mental illnesses reporting the most significant challenges. Self-efficacy and education level showed a positive association with HL across all groups, while social support was positively associated with HL among individuals with chronic mental illness. For respondents with chronic somatic illness, age was negatively associated with HL, whereas social status showed a positive association. Female respondents without chronic illness and those with chronic somatic illness demonstrated higher HL compared to male respondents.

**Conclusion:**

This study advances the understanding of HL among individuals with chronic illness and highlights the need for a greater differentiation among disease groups and HL domains in future research. Particular attention should be paid to people with chronic mental illness, whose lower HL levels increase their vulnerability.

## 1 Introduction

Chronic illnesses continue to dominate the disease spectrum. They are responsible for 74% of all deaths worldwide (1) and 90% of deaths in Europe (2). Cardiovascular diseases are the leading cause of death for years, followed by cancer, chronic respiratory diseases and diabetes (1, 3). Chronic diseases are caused by various endogenous and exogenous factors and manifest themselves in all phases of life, but especially in old age (1, 4, 5). They progress over a long period of time and are usually characterized by changing conditions that constantly present new challenges (6, 7). Health literacy (HL) is needed to cope with these challenges and to adapt to the changing nature of the disease (5, 8).

This also applies to chronic mental illnesses, such as anxiety or mood disorders, which have become more prominent in recent years due to the COVID-19 pandemic. According to the Global Burden of Disease Study, the percentage of people diagnosed with depression increased by 28% worldwide during 2020, the first year of the pandemic; the percentage of anxiety disorders increased by 26% (9, 10). No less important from a public health standpoint: people with severe mental disorders die much earlier (11) from preventable physical health disorders, because mental and physical health are often interrelated (12, 13).

In general, many chronic illnesses—whether mental or somatic—have the untapped, inherent potential to prevent disease and promote health. To better utilize this potential, HL is necessary (14).

In many countries, HL—defined as “people’s knowledge, motivation and competences to access, understand, appraise, and apply health information in order to make judgments and take decisions in everyday life concerning health care, disease prevention and health promotion to maintain or improve quality of life during the life course.” (15, p. 3)—has developed into an important research area with a growing number of studies [e.g., (16–18)]. For the most part, they indicate that the general population’s HL needs improvement (16, 17). This is especially important, as studies have demonstrated that low HL is linked to a wide range of negative outcomes, including unhealthy behaviors, reduced utilization of preventive services, poorer health status and self-management abilities, as well as increased use of the healthcare system—all of which come at significant cost (17, 19–22).

Chronic illnesses also hold significant importance in research on HL. Numerous reviews examine the HL of individuals with chronic conditions, with a focus on cardiovascular diseases (23, 24, 95), diabetes (25), chronic musculoskeletal and rheumatic disorders (26–28), cancer (29, 30), chronic respiratory diseases (31, 32), or kidney disease (33, 34).

However, mostly these studies follow a disease-specific approach and examine the outcomes of functional HL, often focusing on the clinical-medical endpoints of somatic chronic disease [also (35–39)]. There are currently only a few studies that focus on the general population and are based on a multidimensional understanding of HL (15) that includes aspects of health care, prevention and health promotion [also (37, 39)].

With some exceptions (12, 40–42), the same applies to studies on people with mental illness (43). In recent years, a separate field of research has emerged on the topic of mental HL. However, it exists largely separate from the HL debate and focuses more on knowledge about mental illness, its causes, and options for treatment, as well as attitudes and beliefs about illness (44–46). Studies that investigate HL in this population group are largely lacking.

The aim of this article is to contribute to filling this gap. The HL of people with and without chronic illness will be examined comparatively based on data from the Second Health Literacy Survey Germany (HLS-GER 2). A distinction is made between chronic somatic and mental illness, as they embody different types of diseases with different characteristics and progressions. This suggests that the challenges in managing health information also vary. The specific questions are:

- How is the HL of people without chronic illness, with chronic somatic and chronic mental illness distributed across the three HL domains: health care, disease prevention, and health promotion,
- Where do difficulties in managing health-related information arise, and how do they vary between groups, and
- Which socio-demographic, social and psychological factors are related to HL.

## 2 Materials and methods

### 2.1 Data

Data from the HLS-GER 2 were used for the study, which is a quantitative cross-sectional survey of the German-speaking population aged 18 and over, and representative in terms of gender, age, educational level and federal state (18). The survey was conducted by means of paper-assisted personal interviews (PAPI) between December 2019 and January 2020. It was part of the European Health Literacy Population Survey 2019–2021 (HLS_19_) of the WHO Action Network on Measuring Population and Organizational Health Literacy (M-POHL) (17).

For this article, the total sample of the HLS-GER 2 was stratified according to (a) persons without chronic illness, (b) persons with at least one chronic somatic illness, and (c) persons with at least one chronic mental illness. The presence of chronic illness was recorded by means of self-disclosure (“Do you have one or more chronic diseases or long-lasting health problems that have lasted or are expected to last at least 6 months?”) (47). In addition, respondents were asked about their specific disorders based on a list of 25 common somatic and mental health problems. Respondents who stated that they were chronically ill and indicated at least one somatic problem but no mental health problems, were included as cases with at least one chronic somatic illness. Respondents who stated that they suffered from “depression” or “another mental illness” were grouped together as cases with at least one chronic mental illness. It cannot be ruled out that people with chronic mental illnesses also have a chronic somatic illness: Out of the 147 individuals with at least one mental illness, 124 also reported having at least one somatic illness. 23 stated that they were affected solely by at least one mental illness.

### 2.2 Measures

HL was assessed using the latest version of the European Health Literacy Survey Questionnaire (HLS_19_-Q47-DE) with 47 items (17, 18). The instrument measures the subjective difficulties in accessing, understanding, appraising and applying health information, and considers information tasks in the three domains of health care, disease prevention and health promotion (15, 48). It asks how easy or difficult it is to cope with individual information tasks. The items are answered on a 4-point scale (“very easy,” “easy,” “difficult,” “very difficult”). A common HL score (also for the three HL domains) was calculated from the responses (17). The scores range between 0 and 100, with higher values indicating higher HL.

Other variables considered were age, educational level, social status, financial resources, number of illnesses, degree of social support and self-efficacy.

*The educational level* was determined based on the International Standard Classification of Education 2011 (ISCED-11), which defines a total of nine educational levels (49). For the descriptive analysis, levels 0–2, 3 and 4 and levels 5–8 were classified as low, medium and high levels of education, respectively.

*The social status* was surveyed on a scale of 1 (the lowest position in society) to 10 (the highest position in society) by self-assessment (50). Values in the range of 1–4, 5–7 and 8–10 were categorized as low, medium or high social status.

The respondents’ *financial resources* were surveyed using three items on a 4-point scale ranging from “very easy” to “very difficult” (“How easy or difficult is it usually for you to afford medication if needed? To afford medical examinations and treatments, if needed? To pay all bills at the end of the month?”) (17). Financial deprivation was assumed if at least two of the three questions were answered with “difficult” or “very difficult.”

The extent of *social support* was surveyed with the Oslo-3 Social Support Scale (OSSS-3). The scale is made up of three questions on the perceived availability of social support (size of primary support group, interest and involvement of other people and practical help provided by others). The response scores range between 3 and 14 points, with values 3–8, 9–11 and ≥ 12 categorized as low, moderate and strong social support (51).

*Self-efficacy* was considered as a psychological construct. It was measured using the General Self-Efficacy Short Scale (GSE), which uses three questions to assess individual expectations of confidence in dealing with difficulties and obstacles in daily life. A mean scale value was calculated from the given responses (52).

### 2.3 Statistical analyses

All analyses were conducted with the statistical software SPSS version 28.0. The sample characteristics, the items indicated as difficult, and the mean values were calculated using descriptive statistics. The response categories “difficult” and “very difficult” were combined to analyze individual items. Z-tests for column proportions were applied with Bonferroni correction to account for multiple comparisons when comparing categories between groups. Kruskal-Wallis test was used to compare mean values between the groups under consideration. Multiple linear regression models were used to examine the association between variables shown in Table 1 and the HL scores. The dataset used for the univariate and bivariate analyses was weighted for age, educational level, federal state and population density. The regression analyses are based on unweighted data.

**Table 1:** Sample characteristics.

	No chronic illness		At least one chronic somatic illness		At least one chronic mental illness	
	*n*	%	*n*	%	*n*	%
Total	1,026	100	920	100	147	100
*Gender*						
Female	490	47.8	495	53.8	76	51.7
Male	531	51.7	424	46.1	71	48.3
Missing	5	0.5	1	0.1		
*Age (mean; SD)*	(42.6; 16.5)		(59.8; 16.4)		(53.3; 16.3)	
18–29 years	272	26.5	59	6.4	16	10.8
30–45 years	322	31.4	132	14.3	28	19.3
46–64 years	285	27.8	319	34.7	60	41.0
65–75 years	98	9.5	208	22.7	29	19.7
76 years and older	37	3.6	195	21.2	13	9.2
Missing	12	1.1	6	0.7		
*Educational level*						
Low	97	9.5	106	11.5	20	13.9
Medium	617	60.2	531	57.7	83	56.8
High	296	28.9	261	28.4	36	24.8
Missing	15	1.5	22	2.4	7	4.5
*Social status*						
Low	148	14.4	192	20.9	49	33.1
Medium	682	66.5	563	61.2	79	54.0
High	170	16.6	138	15.0	12	8.3
Missing	25	2.5	26	2.9	7	4.6
*Financial deprivation*						
Yes (≥2/3)	117	11.4	181	19.7	45	30.5
No (<2/3)	787	76.7	672	73.1	92	62.5
Missing	122	11.9	67	7.3	10	7.0
*Self-efficacy (mean; SD)*	(4.1; 0.6)		(3.8; 0.8)		(3.6; 0.9)	
*Social support*						
Low	116	11.3	133	14.4	43	29.2
Moderate	465	45.4	442	48.0	67	45.6
Strong	393	38.3	317	34.4	29	20.0
Missing	52	5.0	29	3.1	9.0	5.3
*Number of illnesses*						
One			290	31.5	19	13.2
More than one			630	68.5	127	86.8

SD, standard deviation.

### 2.4 Ethics statement

This study was approved by the Ethics Committee of Bielefeld University (proposal number 2019-103). The study was conducted in accordance with the local legislation and institutional requirements. Written informed consent for participation was not required from the participants or the participants’ legal guardians/next of kin. In accordance with the national legislation, oral consent was obtained.

## 3 Results

For the analysis, a total of 2,093 datasets were used. The gender distribution among the three groups differs only slightly. Respondents without chronic illness are somewhat more likely to be male (51.7%), whereas respondents with at least one chronic somatic or mental illness are more likely to be female (53.8 and 51.7%, respectively). Individuals with chronic somatic (59.8 years) or mental illness (53.3 years) are, on average, older than those without chronic illness (42.6 years). Respondents with chronic somatic illness are generally older than those with chronic mental illness. Detailed information on sample characteristics can be found in Table 1.

### 3.1 Health literacy scores

The mean HL scores range from 55.97 to 63.87 points. Respondents without a chronic illness scored the highest, while those with chronic mental illness scored the lowest. The group with solely chronic somatic illness placed in mid-field, with an average HL score of 60.57.

**Figure 1:**
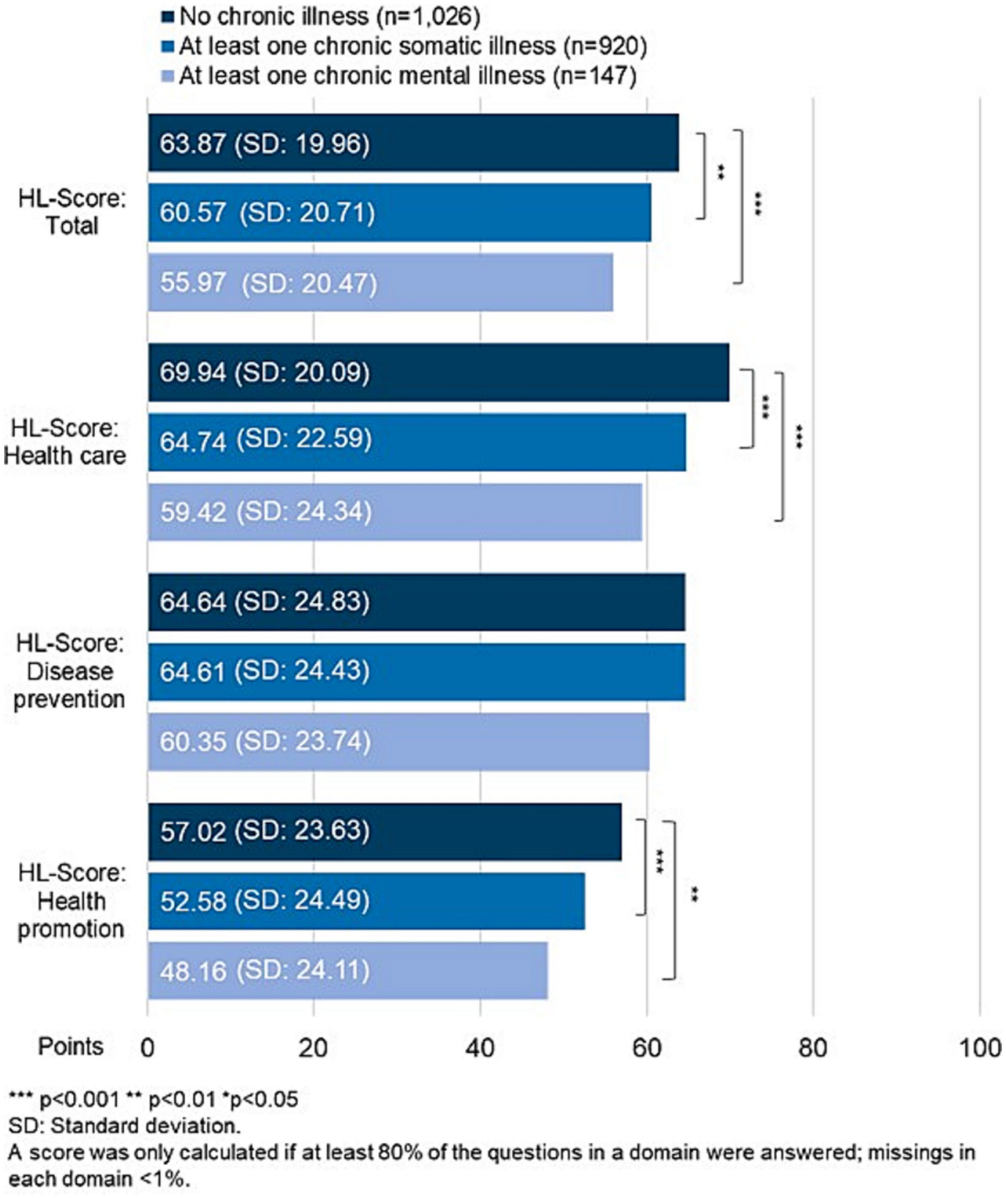
Health literacy scores total and for the three domains health care, disease prevention and health promotion according to the presence of chronic illness.

The pattern is similar in each individual HL domain: Except for disease prevention, the average score values for respondents without a chronic illness are significantly higher than those of the other groups. In contrast, respondents with mental illness have lower scores in all domains than respondents with only somatic illness. However, the differences are not statistically significant. The highest score values are achieved in the domain health care, the lowest score values are achieved in the domain of health promotion (Figure 1).

### 3.2 Difficulties in managing health literacy tasks

**Figure 2:**
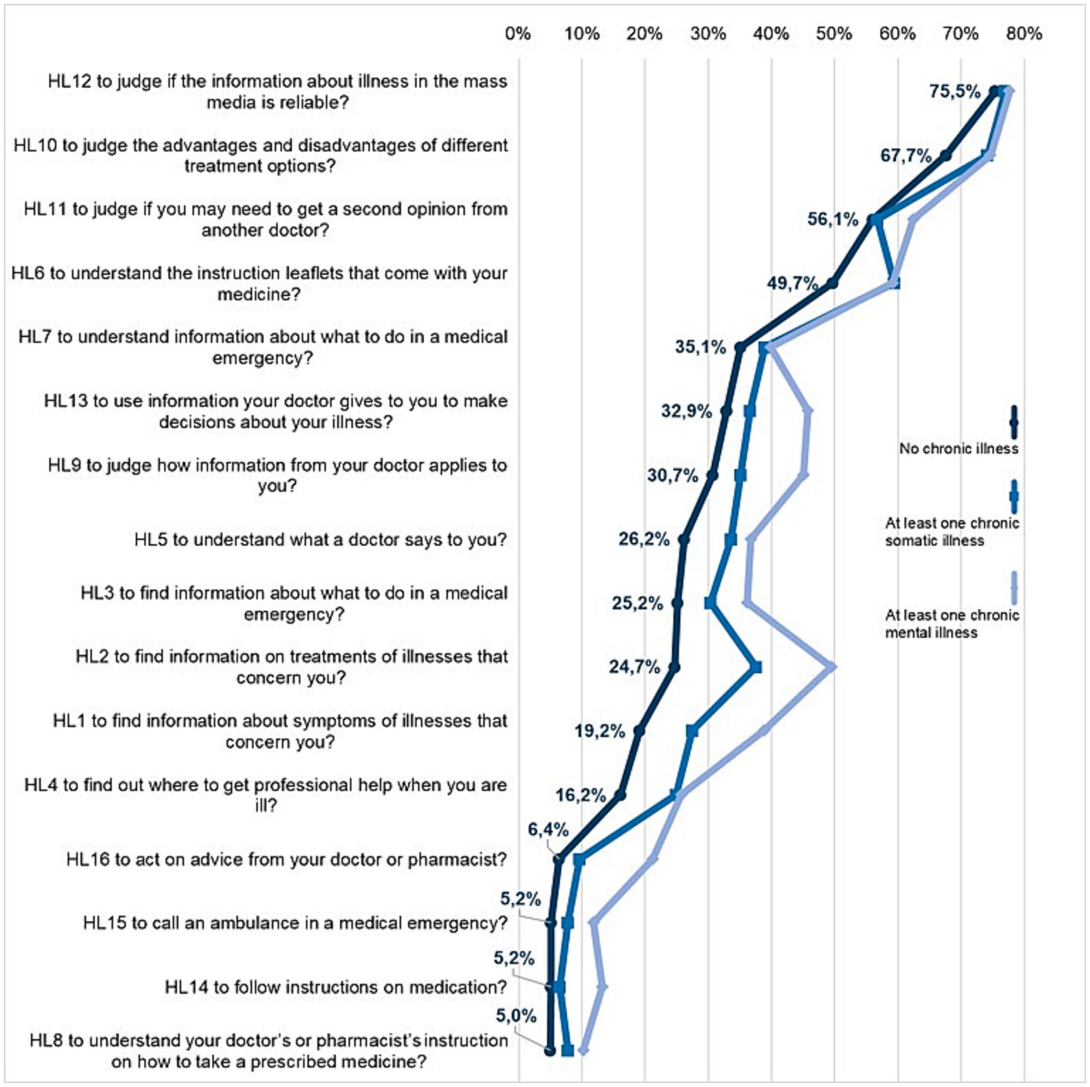
Shares of difficulties (answers “difficult” or “very difficult” combined) for the items in the health literacy domain health care according to the presence of chronic illness.

The tasks in the domain of *health care* are rated the easiest; however, there are also difficulties (Figure 2). All three groups find it the most difficult to judge if health information about illness in the mass media is reliable (HL12, 75.5–77.7% very/difficult), followed by judging the advantages and disadvantages of different treatment options (HL10, 67.7–74.7%).

The third most difficult item received varying responses. For respondents without a chronic illness and with a chronic mental illness, the third most difficult item is to judge whether a second opinion should be obtained from another doctor (HL11, 56.1 and 62.5% respectively). For respondents with a chronic somatic illness, understanding the patient information leaflet is the third most difficult item (HL6, 59.4%) (Figure 2).

Diverging responses are also given for other items: respondents with chronic somatic illness rated 9 out of 16 items (HL1, HL2, HL3, HL4, HL5, HL6, HL8, HL10, HL16) statistically significant (*p* < 0.05) more difficult than respondents without chronic illness. Respondents with chronic mental illness had even greater difficulties with 11 items (HL1, HL2, HL3, HL4, HL5, HL8, HL9 HL13, HL14, HL15, HL16) (*p* < 0.05, Supplementary Table S1). For example, they consider it much more difficult (49.5% very/difficult) to find information on treatments of illnesses than people with somatic illness (37.6%) or those without chronic illness (24.7%) (HL2). This is also true for the task “find information about symptoms of illness that concern you” (HL1): 38.8% of those with mental illness find this task (very) difficult; the numbers are significantly lower for the other groups at 19.2 and 27.5%, respectively.

Respondents consider the domain of *disease prevention* as slightly less difficult—except for the group with chronic mental illnesses. In addition, the answers here are more homogenous. Compared to respondents without chronic illness, only 2 of the 15 items were rated as statistically significant more difficult by respondents with chronic somatic (HL23, HL27) and mental illness (HL25, HL31) (*p* < 0.05, Supplementary Table S1), with particularly large deviations among respondents with chronic mental illness. They find it considerably more difficult to judge when to go to a doctor for a check-up (HL25, 44.6% vs. 32.6 and 28.4% very/difficult) or to decide how to protect from illness using information from the mass media (HL31, 73,9 vs. 62,0% vs. 58,9%).

**Figure 3:**
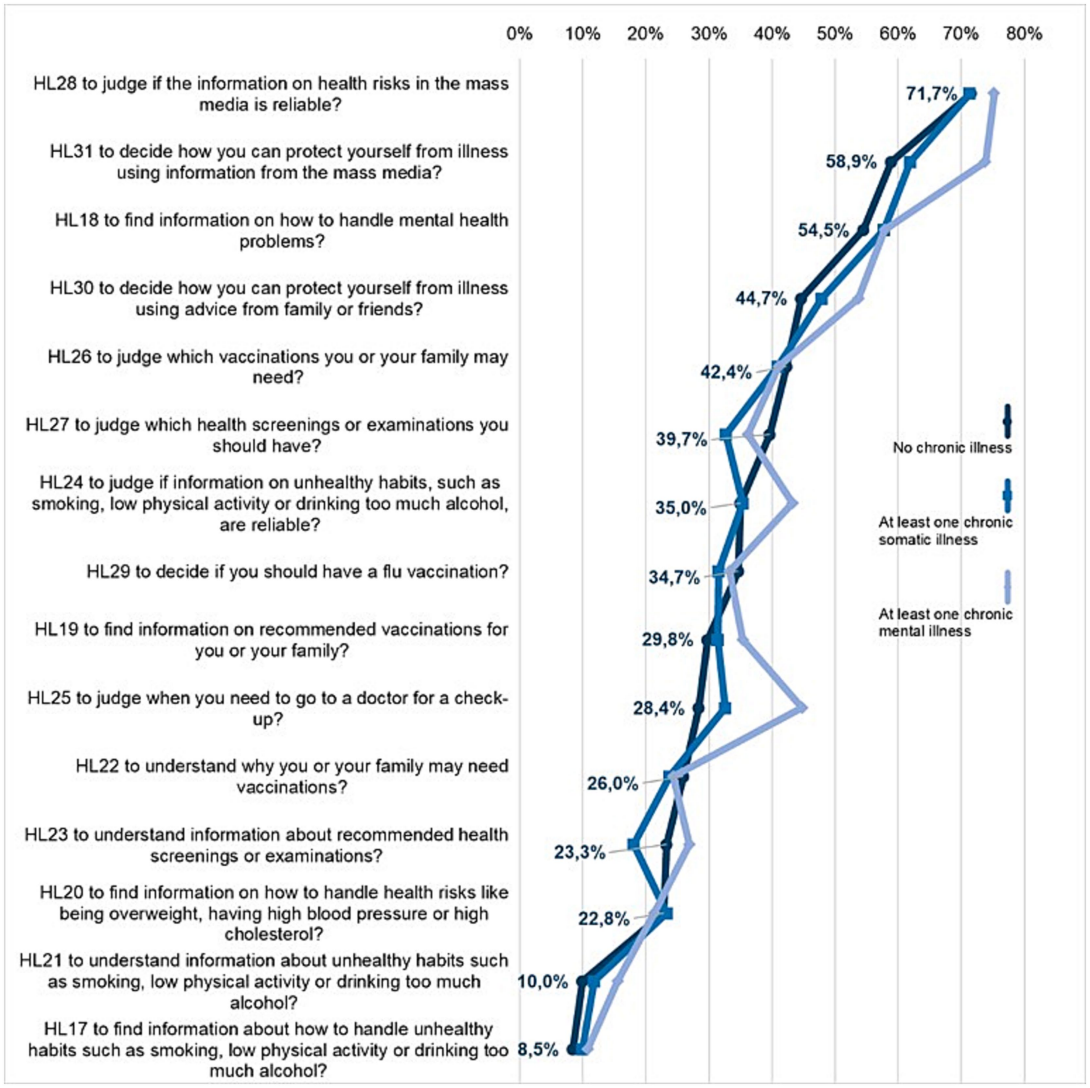
Shares of difficulties (answers “difficult” or “very difficult” combined) for the items in the health literacy domain disease prevention according to the presence of chronic illness.

There are no differences in the order of the three most difficult items. The most difficult items rated by all groups are “to judge if the information on health risks in the mass media is reliable” (HL28, 71.7–75.3%), followed by “to decide how you can protect yourself from illness using information from the mass media” (HL31, 58.9–73.9%) and “to find information on how to handle mental health problems” (HL18, 54.5–57.9%) (Figure 3).

**Figure 4:**
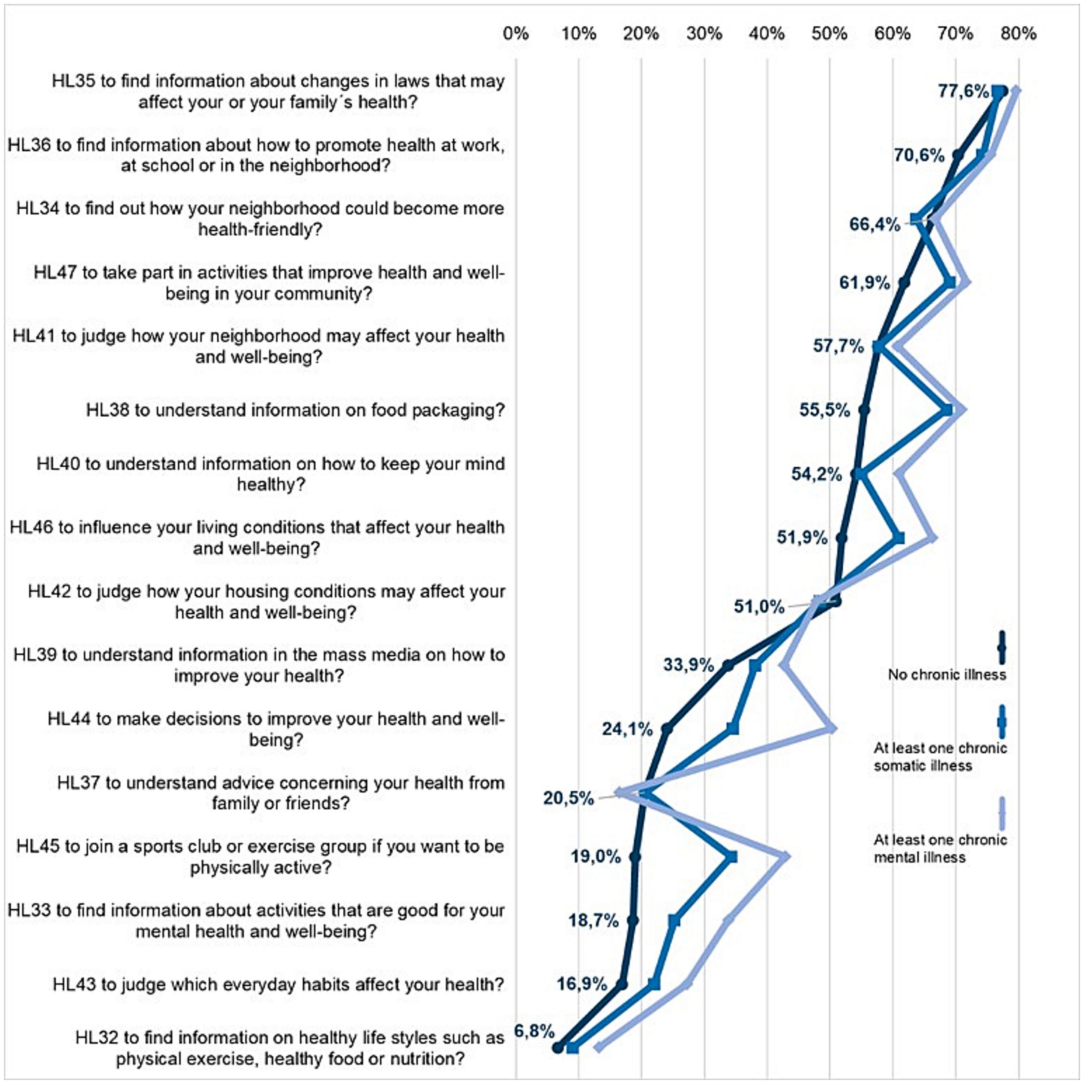
Shares of difficulties (answers “difficult” or “very difficult” combined) for the items in the health literacy domain health promotion according to the presence of chronic illness.

In the domain of *health promotion*, dealing with health information is the most difficult task for all respondents. There are clear differences between respondents without chronic illness and those with chronic somatic and mental illness (Figure 4). A relatively similar response behavior can be observed in the last two groups: 7 of the 16 items total (chronic somatic: HL33, HL38, HL43 HL44, HL45, HL46, HL47; chronic mental: HL32, HL33, HL38, HL43, HL44, HL45, HL46) are answered as statistically significant (*p* < 0.05) more difficult than by respondents without chronic illness (Supplementary Table S1). However, differences can also be observed between the two groups, most notably in item HL44 “to make decisions to improve your health and well-being”: 50.3% of respondents with chronic mental illness rate this as (very) difficult, compared to only 34.6% of those with chronic somatic illness. Significant differences are also evident in item HL33: 33.8% of those with mental illness consider it (very) difficult to find information on activities that have a positive effect on mental health (…) (HL33); for the other two groups, the figures are 18.7 and 25.3%, respectively.

The task “to find information about changes in laws that may affect your or your family’s health” was rated as the most difficult by all three groups (HL35, 77.6–79.7% very/difficult), followed by “to find information about how to promote health at work, at school or in the neighborhood” (HL36, 70.6–75.6%). The task “to find out how your neighborhood could become more health-friendly” ranks third among the most difficult for respondents without and with chronic somatic illness (HL34, 66.4 and 63.7% respectively). For people with chronic mental illness, however, the third most difficult is “to take part in activities that improve health and well-being in your community” (HL47, 71.7%) (Figure 4).

### 3.3 Factors associated with health literacy

Multiple regression models were calculated to analyze which factors are associated with HL (total score). In addition to common socio-demographic and socio-economic variables, social support and self-efficacy were included in the analysis (Table 2).

Controlling for all other variables, the latter shows a positive correlation with HL: in all groups, higher self-efficacy is associated with higher HL (*β* = 0.29, *p* < 0.001; β = 0.38, *p* < 0.001; *β* = 0.27, *p* = 0.003). A relationship between social support and HL was found only among respondents with chronic mental illness. Moderate social support (B = 13.17, <0.001; reference: low) is associated with better HL.

Examining the relationship between gender and HL shows that female respondents without (B = 2.94, *p* = 0.022) and with chronic somatic illness (B = 4.54; *p* < 0.001) rate their HL slightly better than male respondents. There was as negative association between age and HL in respondents with chronic somatic illness (*β* = −0.08, *p* = 0.012).

The educational level was positively linked to HL in all groups. A higher (B = 6.26, *p* = 0.035; B = 17.13, *p* = 0.013) or medium level of education (B = 4.74, *p* = 0.030) is associated with a higher HL; this also applies to a certain extent to social status, which is positively associated with HL among those with chronic somatic illnesses (β = 0.10, *p* = 0.009).

The adjusted *R*^2^ for the calculated models ranges between *R*^2^ = 0.141 und *R*^2^ = 0.245.

Regression models were also calculated for the three HL domains; the results are included in the appendix (Supplementary Tables S2–S4). The observed relationships show similar tendencies as in the overall model but differ in part by domain and group.

**Table 2:** Factors associated with general health literacy scores: results of multiple linear regressions.

	No chronic illness				At least one chronic somatic illness				At least one chronic mental illness			
	B	95%-KI	*p*	*β*	B	95%-KI	*p*	*β*	B	95%-KI	*p*	*β*
Constant	14.77	2.72; 26.85	**0.016**		13.07	2.06; 24.09	**0.020**	**–**	3.62	−24.30; 31.54	0.798	**–**
Age	0.01	−0.06; 0.09	0.725	0.01	−0.10	−0.18; −0.02	**0.012**	−0.08	−0.12	−0.36; 0.12	0.339	−0.08
Gender (ref. male)												
Female	2.94	0.43; 5.46	**0.022**	**–**	4.54	2.12; 6.96	**<0.001**	**–**	0.36	−6.51; 7.22	0.918	**–**
*Level of education* (ref. low)												
Medium	2.52	−2.93; 7.97	0.365		4.74	0.45; 9.02	**0.030**	**–**	20.88	8.22; 33.54	**<0.001**	
High	6.26	0.43; 12.09	**0.035**	**–**	4.49	−0.30; 9.27	0.066	**–**	17.13	3.73; 30.53	**0.013**	**–**
Social status	0.65	−0.33; 1.63	0.195	0.05	1.21	0.31; 2.11	**0.009**	0.10	1.96	−0.44; 4.37	0.109	**–**
Financial deprivation (ref. none)												
Yes	−3.76	−7.94; 0.42	0.078	**–**	−2.36	5.72; 1.01	0.170	**–**	4.38	−3.96; 12.71	0.301	**–**
Social support (ref. low)												
Moderate	−0.13	−4.34; 4.08	0.952	**–**	1.73	−1.88; 5.34	0.346	**–**	13.17	5.32; 21.01	**<0.001**	**–**
Strong	2.77	−1.57; 7.12	0.211	**–**	2.29	−1.54; 6.12	0.240	**–**	8.60	−1.38; 18.59	0.090	**–**
Self-efficacy	9.08	6.90; 11.13	**<0.001**	0.29	9.32	7.68; 10.95	**<0.001**	0.38	6.24	2.12; 10.36	**0.003**	0.27
Number of illnesses (ref. one)												
More than one	**–**	**–**	**–**	**–**	−0.67	−3.34; 1.99	0.619		−1.74	−11.74; 8.27	0.731	**–**
*Adj. R* ^2^	0.141				0.242				0.245			

B, non-standardized coefficient; CI, confidence interval; *β*, standardized coefficient; Adj. *R*^2^, adjusted *R*^2^ (the higher the value, the better the model fits the data); values in bold for *p* < 0.05; Ref., reference group; age: in years, gender: dummy variable ref. male, educational level (ISCED-11): dummy variable ref. low, social status 1 = low to 10 = high, financial deprivation: dummy variable ref. none, social support: dummy variable ref. low, self-efficacy: values from 0 = low to 5 = high, number of illnesses: dummy variable ref. one.

## 4 Discussion

The aim of the study was an in-depth and differentiated analysis of the the HL of chronically ill people and to distinguish between those without chronic illness and people with chronic somatic and mental illness.

1. The key finding is that individuals with chronic mental illness have lower HL scores in all three areas surveyed than respondents without chronic illness and tend to have lower scores than respondents with chronic somatic illness. The few existing studies on the subject come to similar conclusions, although they cannot be compared directly because they are based on different concepts of HL, examine different target groups and use different survey instruments. Nevertheless, they suggest that people with chronic mental illness have a lower HL compared to the general population (12, 40–43, 53). The analysis confirms this finding, and compares it for the first time with chronic somatic diseases among the three domains of health care, disease prevention, and health promotion.

Further studies are needed to substantiate this finding, which is important from a public health perspective. In future, it will also be important to take a more differentiated view on people with chronic illness and to distinguish more clearly between people with chronic somatic and mental illness. Differentiating according to multiple or psychological comorbidities may also prove insightful, as a study by Pedersen et al. (54) shows: As emphasized, it is not uncommon for persons with chronic illness to suffer from several illnesses or from a combination of chronic mental and somatic illnesses that influence each other. This is associated with a lower HL [also (41, 55)]. More differentiated approaches have been called for and have been implemented with other population groups, such as people with migration backgrounds, children and adolescents or older adults (56–60); HL research should place more emphasis on such needs.

The analysis suggests that more attention should be paid to people with chronic mental illness in developing interventions. This is also supported by the steady increase in mental health problems in recent years, which has likely been exacerbated by current crises (COVID-19 pandemic, wars, etc.) and the uncertainties associated with them (61, 62). Persons with chronic mental illness belong to the particularly vulnerable groups with a higher proportion of low HL. This may be partially due to individual symptoms associated with a certain illness (lack of motivation, depression, etc.) which make it difficult or even impossible to deal with information (12). Widescale motivation and intensive empowerment are required to strengthening HL. At the same time, according to the relational model of HL (63), the structural aspect must be critically challenged to determine if sufficient support options and easy-to-find and reliable information even exist for this population group; the findings tend to suggest the opposite. This is exemplified by item HL18 “to find information on how to handle mental health problems?” which was rated as difficult by well over half of all respondents. Germany in particular shows gaps in the outpatient care of persons with mental illness (64, 65), which is often marked by long waiting periods and involved searches on the part of the patient. It can be assumed that this issue at the structural level is also reflected in the respondents’ answers. It also indicates that health care professionals involved in the care of mentally ill patients are an important source of information (66) that, however, cannot be satisfactorily utilized. Therefore, it is important to facilitate access and to also provide alternative sources of mental health-related information.

2. A comparison of the *three domains of HL examined* here shows that people with chronic mental illness find it the most difficult to deal with information in the domain of *health promotion*. In other words, “to lift life above illness” (67) and to do something for their own health and wellbeing beyond coping with their illness seems to be particularly challenging. It indicates that there is not enough accessible information and services available to people with chronic mental illness. At the same time, it underlines the great importance of motivational support and empowerment for this group. However, it should also be mentioned that respondents without and with chronic somatic illness also rate the tasks in the domain of health promotion the most difficult, which indicates that more needs to be done in general to strength HL in this domain.

A look at the individual items in this domain shows just where strengthening is needed. Overall, it is difficult to find information on how health and wellbeing at work, at school, and within the neighborhood can be improved, or, more specifically: to understand information on food packaging, which both groups with chronic illness rated as even more difficult. This shows that promoting HL should begin with everyday life and be geared towards the challenges people face on a daily basis (68–70).

3. HL is better overall in the domain of *disease prevention*, with only slight differences between the three groups. However, it is worth noting that respondents with mental illness also tend to find it more difficult than other groups to manage information in this domain. This is an important indication that steps must be considered when intensifying the prevention of chronic illness, as called for by the WHO (71). Findings show that people with mental illness should be given more consideration as a target group. Also notable is the item rated as most difficult in this domain, which is dealing with the media. All groups find it difficult to assess information from the media about health risks or to use information to make decisions about disease prevention. Respondents with chronic mental illness find this more difficult than the other two groups, which is also important for developing interventions. Improving the quality of health-related information available from the media, with its mix of serious and dubious sources, is undoubtedly one of the most important tasks ahead, with current developments in artificial intelligence (AI) only emphasizing its relevance (72, 73).

4. All groups rate dealing with information in the domain *health care* as the easiest, as studies on general HL confirm (18, 74, 75). What is interesting is that the level of difficulty is in keeping with the pattern of the overall score: people without chronic illness have the least difficulty with these tasks, while people with chronic mental illness have the most difficulty. At the same time, the response pattern of people with chronic somatic and chronic mental illness differs significantly from that of the group without chronic illness. This is also worth noting for the development of interventions—especially from a target group perspective.

When examining the most difficult items, it is striking that all groups rate tasks that focus on evaluating information the most difficult. Other HL studies support this finding; they also show that the assessment of media or digital information is particularly difficult (76–79). Mantell et al. (12) arrive at a similar finding in their study on HL in predominantly younger people with mental illness. Improving the quality and assessability of information is therefore an important challenge that is particularly relevant in times of an infodemic (80, 81). Support to assess and interpret health information is also urgently needed, whether through counseling or through better communication by health care professionals—who continue to be an important resource—in explaining and conveying health information (82, 83).

5. The question of which *influencing factors* are associated with HL within the three groups was also investigated. Emphasis was placed on social support and self-efficacy. To our knowledge, this is the first study to examine this comparatively for respondents without and with chronic somatic and chronic mental illnesses. It was shown that better self-efficacy is associated with a higher HL in all three groups, which demonstrates its importance to HL in general; other studies confirm this (56, 84, 85). The literature also considers the degree of social support to be important for HL in coping with chronic illness [e.g., (86–88)]. This analysis shows that social support and HL are associated in the group of people with chronic mental illness. It can be assumed that the social environment plays an important role in the processing of information for people with mental illness. However, social support is less pronounced than in the other two groups, which shows the potential of building and strengthening networks, which would also benefit HL.

The socio-demographic and economic factors that were examined present a mixed bag of findings. For example, age, which was identified as a relevant predictor of HL in previous studies (60, 89, 90), is only associated with HL in the group of people with chronic somatic illnesses. This also applies to social status but not to educational level. A higher level of education is associated with higher HL in all groups. Other studies support this finding (17, 18, 89, 91). In the present analysis, this association is clearly visible in the group of people with chronic mental illness. While this result should be interpreted cautiously due to the small sample sizes within the individual groups, it indicates the importance of this group’s educational level. Further studies on this association are recommended.

### 4.1 Limitations

It should be noted that HL was collected using a self-assessment tool that measures subjective difficulties in dealing with health information that do not necessarily correspond to the respondents’ actual abilities. The advantages and disadvantages of these instruments have already been described in detail in the literature (92, 93). In addition, the underlying survey is a cross-sectional study that does not allow any statements to be made about the causality of the findings. Another limiting factor is that although the HLS-GER 2 survey can be considered representative of the adult population in Germany in terms of gender combined with age and level of education, it is to be expected that the group of people with chronic illness differs from the population of chronically ill people in Germany. This may apply especially to respondents with mental illness, who are only represented by 147 people in the sample and who have mostly indicated “depression” as their illness. This limits the generalizability of the findings, even though depression is one of the most common mental illnesses in Germany (94). It should also be considered that the presence of a chronic illness is based on self-statements, which is a common procedure in population surveys (4, 47). Furthermore, the HLS-GER 2 study was conducted back in 2019/2020. Although it is possible that HL has changed over time, it is hypothesized that the observable trends have remained consistent. This should be examined in future research. Overall, it is recommended that in addition to considering social and psychological aspects, future research should also investigate further determinants of HL to increase the explained variability in the models. In this regard, beside personal, also structural factors should be included, as this aspect has so far hardly been considered in population-based analyses of HL.

## 5 Conclusion

Reducing chronic illness is a major global public health challenge. HL is necessary to prevent disease and reduce the probability of occurrence, as well as to manage diseases competently and strengthen health resources and potential. To promote HL in a targeted manner, it is important to measure HL in the general population. With this analysis, it was demonstrated how promising it is to differentiate between chronic somatic and mental illness, because the difficulty profiles differ significantly among both groups. The findings also show that the full utilization of preventive and health-promoting potential represents a great obstacle for people with mental illness, which may partially be due to disease-specific causes but also to social and systemic factors.

This points to several noteworthy particularities in this group and indicates that people with chronic mental illness should be given more consideration in the future, both in research and intervention development. The results of the present study can encourage practitioners in psychological care, as well as researchers, to engage more deeply with the HL of individuals with chronic mental illness. At the same time, the results provide guidance to decision-makers on where efforts in promoting HL can be focused. In addition to incorporating HL aspects (e.g., promoting critical HL) into psycho-educational interventions, the emphasis should be on improving the information environment of the health care system and to ensure that health information is specifically tailored to the target group. This would help reduce existing inequalities.

## Data Availability

The data analyzed in this study is subject to the following licenses/restrictions: further inquiries on raw data can be directed to the corresponding author. Requests to access these datasets should be directed to lennert.griese@uni-bielefeld.de.
