# Supplementary material for: Health literacy and chronic disease: a comparison of somatic and mental illness
Source: Front Public Health. 2025 Feb 18;13:1523723. doi: 10.3389/fpubh.2025.1523723 (PMC11876042; doi:10.3389/fpubh.2025.1523723)
Supplement: Supplementary file 1 [file Table_1.docx]

Supplementary Material

# Supplementary Tables

Supplementary table 1 Items for the three health literacy domains ranked by difficulties according to the presence of chronic illness

| Health care | No chronic illness (A) | At least on chronic somatic illness (B) | At least one chronic mental illness (C) | Comparison of column proportions |
| --- | --- | --- | --- | --- |
| HL12 to judge if the information about illness in the mass media is reliable? | 75.5% | 77.1% | 77.7% | n.s. |
| HL10 to judge the advantages and disadvantages of different treatment options? | 67.7% | 74.3% | 74.7% | A vs. B** |
| HL11 to judge if you may need to get a second opinion from another doctor? | 56.1% | 56.8% | 62.5% | n.s. |
| HL6 to understand the instruction leaflets that come with your medicine? | 49.7% | 59.4% | 59.2% | A vs. B*** |
| HL7 to understand information about what to do in a medical emergency? | 35.1% | 39.0% | 39.9% | n.s. |
| HL13 to use information your doctor gives to you to make decisions about your illness? | 32.9% | 36.6% | 45.8% | A vs. C** |
| HL9 to judge how information from your doctor applies to you? | 30.7% | 35.1% | 45.1% | A vs. C** |
| HL5 to understand what a doctor says to you? | 26.2% | 33.6% | 36.8% | A vs. B**. A vs. C* |
| HL3 to find information about what to do in a medical emergency? | 25.2% | 30.4% | 36.2% | A vs. B*. A vs. C* |
| HL2 to find information on treatments of illnesses that concern you? | 24.7% | 37.6% | 49.5% | A vs. B***. A vs. C***. B vs. C* |
| HL1 to find information about symptoms of illnesses that concern you? | 19.2% | 27.5% | 38.8% | A vs. B***. A vs. C***. B vs. C* |
| HL4 to find out where to get professional help when you are ill? | 16.2% | 24.9% | 25.8% | A vs. B***. A vs. C* |
| HL16 to act on advice from your doctor or pharmacist? | 6.4% | 9.7% | 21.2% | A vs. B*. A vs. C***. B vs. C*** |
| HL15 to call an ambulance in a medical emergency? | 5.2% | 7.8% | 11.9% | A vs. C** |
| HL14 to follow instructions on medication? | 5.2% | 6.5% | 13.3% | A vs. C***. B vs. C* |
| HL8 to understand your doctor’s or pharmacist’s instruction on how to take a prescribed medicine? | 5.0% | 7.8% | 10.3% | A vs. B*. A vs. C* |
|  |  |  |  |  |
| Disease Prevention | No chronic illness (A) | At least on chronic somatic illness (B) | At least one chronic mental illness (C) | Comparison of column proportions |
| HL28 to judge if the information on health risks in the mass media is reliable? | 71.7% | 71.5% | 75.3% | n.s. |
| HL31 to decide how you can protect yourself from illness using information from the mass media? | 58.9% | 62.0% | 73.9% | A vs. C**. B vs. C* |
| HL18 to find information on how to handle mental health problems? | 54.5% | 57.8% | 57.9% | n.s. |
| HL30 to decide how you can protect yourself from illness using advice from family or friends? | 44.7% | 47.9% | 53.7% | n.s. |
| HL26 to judge which vaccinations you or your family may need? | 42.4% | 40.9% | 41.1% | n.s. |
| HL27 to judge which health screenings or examinations you should have? | 39.7% | 32.7% | 36.2% | A vs. B** |
| HL24 to judge if information on unhealthy habits. such as smoking. low physical activity or drinking too much alcohol. are reliable? | 35.0% | 35.4% | 43.3% | n.s. |
| HL29 to decide if you should have a flu vaccination? | 34.7% | 31.6% | 33.3% | n.s. |
| HL19 to find information on recommended vaccinations for you or your family? | 29.8% | 31.4% | 35.4% | n.s. |
| HL25 to judge when you need to go to a doctor for a check-up? | 28.4% | 32.6% | 44.8% | A vs. C***. B vs. C* |
| HL22 to understand why you or your family may need vaccinations? | 26.0% | 23.8% | 24.4% | n.s. |
| HL23 to understand information about recommended health screenings or examinations? | 23.3% | 18.1% | 26.9% | A vs. B*. B vs. C* |
| HL20 to find information on how to handle health risks like being overweight. having high blood pressure or high cholesterol? | 22.8% | 23.3% | 21.3% | n.s. |
| HL21 to understand information about unhealthy habits such as smoking. low physical activity or drinking too much alcohol? | 10.0% | 11.8% | 15.6% | n.s. |
| HL17 to find information about how to handle unhealthy habits such as smoking. low physical activity or drinking too much alcohol? | 8.5% | 10.0% | 10.8% | n.s. |
|  |  |  |  |  |
| Health Promotion | No chronic illness (A) | At least on chronic somatic illness (B) | At least one chronic mental illness (C) | Comparison of column proportions |
| HL35 to find information about changes in laws that may affect your or your family´s health? | 77.6% | 76.8% | 79.7% | n.s. |
| HL36 to find information about how to promote health at work. at school or in the neighborhood? | 70.6% | 74.4% | 75.6% | n.s. |
| HL34 to find out how your neighborhood could become more health-friendly? | 66.4% | 63.7% | 66.7% | n.s. |
| HL47 to take part in activities that improve health and well-being in your community? | 61.9% | 69.0% | 71.7% | A vs. B** |
| HL41 to judge how your neighborhood may affect your health and well-being? | 57.7% | 57.8% | 60.7% | n.s. |
| HL38 to understand information on food packaging? | 55.5% | 68.6% | 71.0% | A vs. B***. A vs. C** |
| HL40 to understand information on how to keep your mind healthy? | 54.2% | 55.1% | 61.0% | n.s. |
| HL46 to influence your living conditions that affect your health and well-being? | 51.9% | 60.9% | 66.3% | A vs. B***. A vs. C** |
| HL42 to judge how your housing conditions may affect your health and well-being? | 51.0% | 48.4% | 47.9% | n.s. |
| HL39 to understand information in the mass media on how to improve your health? | 33.9% | 38.1% | 42.6% | n.s. |
| HL44 to make decisions to improve your health and well-being? | 24.1% | 34.6% | 50.3% | A vs. B***. A vs. C***. B vs. C** |
| HL37 to understand advice concerning your health from family or friends? | 20.5% | 20.6% | 16.5% | n.s. |
| HL45 to join a sports club or exercise group if you want to be physically active? | 19.0% | 34.3% | 42.9% | A vs. B***; A vs. C*** |
| HL33 to find information about activities that are good for your mental health and well-being? | 18.7% | 25.3% | 33.8% | A vs. B**. A vs. C *** |
| HL43 to judge which everyday habits affect your health? | 16.9% | 22.0% | 27.3% | A vs. B*. A vs. C** |
| HL32 to find information on healthy life styles such as physical exercise. healthy food or nutrition? | 6.8% | 9.1% | 13.2% | A vs. C* |
| This table shows the items for the three health literacy domains health care, disease prevention, and health promotion ranked by difficulties (percentages for “difficult” and “very difficult” combined) according to the presence of chronic illness.  *** p<0.001 ** p<0.01 *p<0.05  n.s not significant | | | | |

Supplementary table 2: Factors associated with health literacy scores (health care): Results of multiple linear regressions

|  | No chronic illness | At least one chronic somatic illness | At least one chronic mental illness |
| --- | --- | --- | --- |

|  | **B** | **95 %-KI** | **p** | **β** | **B** | **95 %-KI** | **p** | **β** | **B** | **95 %-KI** | **p** | **Β** |
| --- | --- | --- | --- | --- | --- | --- | --- | --- | --- | --- | --- | --- |
| Constant | 21.80 | 9.84; 33.76 | **<0.001** |  | 18.50 | 6.08; 30.92 | **0.004** |  | 7.32 | -25.69; 40.33 | 0.661 |  |
| Age | -0.01 | -0.08; 0.07 | 0.865 | -0.01 | -0.09 | -0.18; 0.003 | 0.058 | -0.06 | -0.14 | -0.43; 0.14 | 0.323 | 0.09 |
| Gender (ref. male) |  |  |  |  |  |  |  |  |  |  |  |  |
| Female | 1.02 | -1.48; 3.51 | 0.424 | 0.03 | 2.99 | 0.25; 5.73 | **0.032** | 0.07 | -1.11 | -9.23; 7.01 | 0.787 | -0.02 |
| *Level of education* (ref. low) |  |  |  |  |  |  |  |  |  |  |  |  |
| Medium | 4.19 | -1.22; 9.59 | 0.129 | 0.11 | 3.52 | -1.31; 8.35 | 0.15 | 0.08 | 20.73 | 5.77; 35.69 | **0.007** | 0.43 |
| High | 6.43 | 0.65; 12.22 | **0.029** | 0.16 | 3.58 | -1.82; 8.97 | 0.193 | 0.08 | 13.13 | -2.71; 28.96 | 0.103 | 0.26 |
| Social status | 0.81 | -0.17; 1.78 | 0.104 | 0.06 | 0.94 | -0.08; 1.96 | 0.070 | 0.07 | 1.97 | -0.87; 4.82 | 0.173 | 0.13 |
| Financial deprivation (ref. none) |  |  |  |  |  |  |  |  |  |  |  |  |
| Yes | -4.19 | -8.33; -0.05 | **0.048** | -0.07 | -2.80 | -6.60; 1.00 | 0.148 | -0.05 | 3.05 | -6.81; 12.90 | 0.541 | 0.06 |
| Social support (ref. low) |  |  |  |  |  |  |  |  |  |  |  |  |
| Moderate | -1.95 | -6.13; 2.22 | 0.359 | -0.05 | 2.84 | -1.23; 6.91 | 0.172 | 0.06 | 11.51 | 2.24; 20.79 | **0.015** | 0.25 |
| Strong | 0.26 | -4.05; 4.58 | 0.904 | 0.01 | 4.03 | -0.29; 8.35 | 0.067 | 0.09 | 6.81 | -4.99; 18.61 | 0.255 | 0.12 |
| Self-efficacy | 9.64 | 7.54; 11.74 | **<0.001** | 0.31 | 10.01 | 8.17; 11.86 | **<0.001** | 0.37 | 7.14 | 2.27; 12.01 | **0.004** | 0.27 |
| Number of illnesses (ref. one) |  |  |  |  |  |  |  |  |  |  |  |  |
| More than one | - | - | - | - | -1.65 | -4.65; 1.35 | 0.281 | -0.04 | 1.54 | -10.28; 13.36 | 0.797 | 0.02 |
| ***Adj. R²*** | 0.145 |  |  |  | 0.214 |  |  |  | 0.184 |  |  |  |
| B: non-standardized coefficient; CI: confidence interval; β: standardized coefficient; Adj. R^2^: adjusted R^2^ (the higher the value, the better the model fits the data); values in bold for p<0.05; Ref.: reference group; age: in years, gender: dummy variable ref. male, educational level (ISCED-11): dummy variable ref. low, social status: 1=low to 10=high, financial deprivation: dummy variable ref. none, social support: dummy variable ref. low, self-efficacy: values from 0=low to 5=high, number of illnesses: dummy variable ref. one. | | | | | | | | | | | | |

Supplementary table 3: Factors associated with health literacy scores (disease prevention): Results of multiple linear regressions

|  | No chronic illness | At least one chronic somatic illness | At least one chronic mental illness |
| --- | --- | --- | --- |

|  | **B** | **95 %-KI** | **p** | **β** | **B** | **95 %-KI** | **p** | **β** | **B** | **95 %-KI** | **p** | **Β** |
| --- | --- | --- | --- | --- | --- | --- | --- | --- | --- | --- | --- | --- |
| Constant | 10.81 | -4.63; 26.26 | 0.170 |  | 20.72 | 6.70; 34.73 | **0.004** |  | 13.68 | -21.45; 48.82 | 0.442 |  |
| Age | 0.09 | -0.01; 0.19 | 0.074 | 0.06 | -0.05 | -0.16; 0.05 | 0.297 | -0.04 | -0.05 | -0.35; 0.25 | 0.738 | -0.03 |
| Gender (ref. male) |  |  |  |  |  |  |  |  |  |  |  |  |
| Female | 5.49 | 2.27; 8.72 | **<0.001** | 0.11 | 4.26 | 1.17; 7.35 | **0.007** | 0.09 | 2.88 | -5.76; 11.52 | 0.511 | 0.06 |
| *Level of education* (ref. low) |  |  |  |  |  |  |  |  |  |  |  |  |
| Medium | 2.74 | -4.24; 9.72 | 0.441 | 0.06 | 2.75 | -2.72; 8.23 | 0.324 | 0.06 | 17.40 | 1.47; 33.32 | **0.033** | 0.36 |
| High | 6.64 | -0.83; 14.11 | 0.081 | 0.13 | 1.72 | -4.40; 7.83 | 0.582 | 0.03 | 15.86 | -0.10; 32.72 | 0.065 | 0.31 |
| Social status | 0.14 | -1.11; 1.40 | 0.824 | 0.01 | 1.38 | 0.23; 2.53 | **0.019** | 0.10 | 2.79 | 0.25; 5.81 | 0.072 | 0.18 |
| Financial deprivation (ref. none) |  |  |  |  |  |  |  |  |  |  |  |  |
| Yes | -2.77 | -8.12; 2.58 | 0.310 | 0.04 | 2.75 | -7.03; 1.54 | 0.209 | 0.05 | 2.21 | -8.28; 12.71 | 0.677 | 0.04 |
| Social support (ref. low) |  |  |  |  |  |  |  |  |  |  |  |  |
| Moderate | 0.12 | -5.27; 5.52 | 0.965 | 0.002 | 1.25 | 3.36; 5.85 | 0.595 | 0.03 | 11.78 | 1.91; 21.66 | **0.020** | 0.25 |
| Strong | 3.27 | -2.30; 8.83 | 0.250 | 0.07 | -0.21 | -5.09; 4.68 | 0.934 | -0.004 | 2.07 | -10.50; 14.63 | 0.745 | 0.04 |
| Self-efficacy | 9.01 | 6.30; 11.73 | **<0.001** | 0.24 | 8.01 | 5.93; 10.10 | **<0.001** | 0.28 | 3.23 | -1.95; 8.42 | 0.219 | 0.12 |
| Number of illnesses (ref. one) |  |  |  |  |  |  |  |  |  |  |  |  |
| More than one | - | - | - | - | 1.12 | -2.27; 4.51 | 0.518 | 0.02 | -4.71 | 17.29; 7.88 | 0.460 | -0.07 |
| ***Adj. R²*** | 0.089 |  |  |  | 0.115 |  |  |  | 0.103 |  |  |  |
| B: non-standardized coefficient; CI: confidence interval; β: standardized coefficient; Adj. R^2^: adjusted R^2^ (the higher the value, the better the model fits the data); values in bold for p<0.05; Ref.: reference group; age: in years, gender: dummy variable ref. male, educational level (ISCED-11): dummy variable ref. low, social status: 1=low to 10=high, financial deprivation: dummy variable ref. none, social support: dummy variable ref. low, self-efficacy: values from 0=low to 5=high, number of illnesses: dummy variable ref. one. | | | | | | | | | | | | |

Supplementary table 4: Factors associated with health literacy scores (health promotion): Results of multiple linear regressions

|  | No chronic illness | At least one chronic somatic illness | At least one chronic mental illness |
| --- | --- | --- | --- |

|  | **B** | **95 %-KI** | **p** | **β** | **B** | **95 %-KI** | **p** | **β** | **B** | **95 %-KI** | **p** | **Β** |
| --- | --- | --- | --- | --- | --- | --- | --- | --- | --- | --- | --- | --- |
| Constant | 9.73 | -5.09; 24.55 | 0.198 |  | 0.82 | -12.69; 14.34 | 0.905 |  | -3.42 | -37.25; 30.40 | 0.841 |  |
| Age | -0.03 | -0.13; 0.06 | 0.51 | -0.02 | -0.16 | -0.26; -0.06 | **0.001** | -0.11 | -0.17 | 0.46; 0.11 | 0.236 | -0.10 |
| Gender (ref. male) |  |  |  |  |  |  |  |  |  |  |  |  |
| Female | 2.44 | -0.65; 5.52 | 0.122 | 0.05 | 6.29 | 3.31; 9.26 | **<0.001** | 0.13 | -1.63 | -9.87; 6.61 | 0.696 | -0.03 |
| *Level of education* (ref. low) |  |  |  |  |  |  |  |  |  |  |  |  |
| Medium | 0.61 | -6.07; 7.29 | 0.858 | 0.013 | 7.74 | 2.49; 12.99 | **0.004** | 0.16 | 23.99 | 8.91; 39.08 | **0.002** | 0.47 |
| High | 5.42 | -1.74; 12.57 | 0.138 | 0.11 | 7.82 | 1.96; 13.68 | **0.009** | 0.15 | 22.71 | 6.74; 38.68 | **0.005** | 0.42 |
| Social status | 1.10 | -0.10; 2.30 | 0.073 | 0.07 | 1.36 | 0.26; 2.47 | **0.016** | 0.09 | 0.90 | -1.99; 3.78 | 0.540 | 0.06 |
| Financial deprivation (ref. none) |  |  |  |  |  |  |  |  |  |  |  |  |
| Yes | -3.84 | -8.96; 1.29 | 0.142 | -0.05 | -1.97 | -6.10; 2.17 | 0.350 | -0.03 | 5.64 | -4.50; 15.78 | 0.273 | 0.10 |
| Social support (ref. low) |  |  |  |  |  |  |  |  |  |  |  |  |
| Moderate | 1.20 | -3.96; 6.37 | 0.648 | 0.03 | 1.41 | -3.03; 5.85 | 0.532 | 0.03 | 17.70 | 8.23; 27.17 | **<0.001** | 0.36 |
| Strong | 4.89 | -0.44; 10.22 | 0.072 | 0.10 | 3.08 | -1.63; 7.78 | 0.199 | 0.06 | 18.38; | 6.37; 30.39 | **0.003** | 0.30 |
| Self-efficacy | 8.59 | 5.99; 11.20 | **<0.001** | 0.23 | 9.68 | 7.66; 11.69 | **<0.001** | 0.33 | 7.57 | 2.62; 12.52 | **0.003** | 0.27 |
| Number of illnesses (ref. one) |  |  |  |  |  |  |  |  |  |  |  |  |
| More than one | - | - | - | - | -1.44 | -4.70; 1.82 | 0.386 | -0.03 | -2.69 | -14.62; 9.22 | 0.654 | -0.04 |
| ***Adj. R²*** | 0.108 |  |  |  | 0.215 |  |  |  | 0.260 |  |  |  |
| B: non-standardized coefficient; CI: confidence interval; β: standardized coefficient; Adj. R^2^: adjusted R^2^ (the higher the value, the better the model fits the data); values in bold for p<0.05; Ref.: reference group; age: in years, gender: dummy variable ref. male, educational level (ISCED-11): dummy variable ref. low, social status: 1=low to 10=high, financial deprivation: dummy variable ref. none, social support: dummy variable ref. low, self-efficacy: values from 0=low to 5=high, number of illnesses: dummy variable ref. one. | | | | | | | | | | | | |
